# Etiology and clinical characteristics of SARS-CoV-2 and other human coronaviruses among children in Zhejiang Province, China 2017–2019

**DOI:** 10.1186/s12985-021-01562-8

**Published:** 2021-04-30

**Authors:** Yanjun Zhang, Lingxuan Su, Yin Chen, Sicong Yu, Dan Zhang, Haiyan Mao, Lei Fang

**Affiliations:** 1 Department of Laboratory, School of Medical Technology, Zhejiang Chinese Medical University, Hangzhou, 310053 China; 2 Zhejiang Provincial Center of Disease Control and Prevention, 3399 Bincheng Road, Hangzhou, 310051 China; 3 Department of Laboratory Medicine, The First Affiliated Hospital, Zhejiang University School of Medicine, Hangzhou, 310000 China

**Keywords:** Human coronavirus, Hospitalized children, Phylogenic analysis, Acute respiratory infection, SARS-CoV-2

## Abstract

**Background:**

A novel coronavirus (SARS-CoV-2) emerging has put global public health institutes on high alert. Little is known about the epidemiology and clinical characteristics of human coronaviruses infections in relation to infections with other respiratory viruses.

**Methods:**

From February 2017 to December 2019, 3660 respiratory samples submitted to Zhejiang Children Hospital with acute respiratory symptoms were tested for four human coronaviruses RNA by a novel two-tube multiplex reverse transcription polymerase chain reaction assays. Samples were also screened for the occurrence of SARS-CoV-2 by reverse transcription-PCR analysis.

**Results:**

Coronavirus RNAs were detected in 144 (3.93%) specimens: HCoV-HKU1 in 38 specimens, HCoV-NL63 in 62 specimens, HCoV-OC43 in 38 specimens and HCoV-229E in 8 specimens. Genomes for SARS-CoV-2 were absent in all specimens by RT-PCR analysis during the study period. The majority of HCoV infections occurred during fall months. No significant differences in gender, sample type, year were seen across species. 37.5 to 52.6% of coronaviruses detected were in specimens testing positive for other respiratory viruses. Phylogenic analysis identified that Zhejiang coronaviruses belong to multiple lineages of the coronaviruses circulating in other countries and areas.

**Conclusion:**

Common HCoVs may have annual peaks of circulation in fall months in the Zhejiang province, China. Genetic relatedness to the coronaviruses in other regions suggests further surveillance on human coronaviruses in clinical samples are clearly needed to understand their patterns of activity and role in the emergence of novel coronaviruses.

**Supplementary information:**

The online version contains supplementary material available at 10.1186/s12985-021-01562-8.

## Introduction

Human coronaviruses (HCoVs) are common viruses that cause mild to severe respiratory infections. To date, there are seven CoVs have been confirmed to infect human, including two α-CoVs (HCoV-229E and HCoV-NL63) and five β -CoVs [HCoV-HKU1, HCoV-OC43, severe acute respiratory syndrome coronavirus (SARS-CoV) and SARS-CoV-2, and Middle East respiratory syndrome coronavirus (MERS-CoV)] [1]. The ongoing pandemic of COVID-19 offers the most recent example of the tragedy when highly pathogenic coronaviruses emerge in the human population [2–4]. As of 23 March 2021, approximately 25 million cases have been reported to the WHO accounting for a 2.2% case fatality rate. Over the last two decades, the emergence of Severe Acute Respiratory Syndrome Coronavirus (SARS-CoV) has been associated with severe atypical pneumonia and caused 774 deaths [5]. In 2012, a man in Saudi Arabia who infected by Middle East Respiratory Syndrome Coronavirus (MERS-CoV) died of acute pneumonia and renal failure [6]. The continuing spread of MERS-CoV resulted in unprecedented outbreaks and high case-fatality rates.

HCoV-NL63, HCoV-HKU1, HCoV-229E, and HCoV-OC43 circulate worldwide and cause a range of respiratory symptoms [7]. These four HCoVs result in 30% of common colds and acute respiratory infections such as pneumonia and bronchiolitis, especially in immunocompromised children [8, 9]. Lower respiratory tract infections are considered as the most common cause of death among children and are responsible for 15% mortality rate of children under 5 years of age globally [10, 11]. HCoVs pose serious threats to children health as they are associated with acute upper or lower respiratory tract infections [12, 13]. Although HCoVs are distributed globally, the predominant species may vary by region or year. Moreover, a notable feature of COVID-19 pandemic is the relative paucity of published report that describes the potential impact of SARS-CoV-2 on children with acute respiratory infection [14]. However, the prevalence and clinical profiles of HCoVs have been largely underdetermined and no studies described circulation of SARS-CoV-2 and four common HCoVs across multiple years in Zhejiang province, China.

Considering the discovery of novel SARS-CoV-2, we retrospectively investigated the role of SARS-CoV-2 and common human coronaviruses (HCoV-229E, HCoV-OC43, HCoV-NL63 and HCoV-HKU1) in childhood respiratory infections in Zhejiang province, China, and results were compared to data for other viral respiratory pathogens including influenza A and B, parainfluenza, respiratory syncytial virus, rhinovirus, and adenovirus.

## Materials and methods

### Ethical approval statement

The studies involving human participants were reviewed and approved by Zhejiang Provincial Center for Disease Control and Prevention of ethics committee. Children’s parents/guardians have been notified the purpose of the study and the right to keep information confidential. Children’s parents/guardians provided their written informed consent to participate in this study.

### Sample collection

A total of 3660 retrospective respiratory samples were collected from children (age, ≤ 18 years) with acute respiratory tract infections who admitted to Children’s Hospital of Zhejiang University from 2017 to 2019. Written informed consent was obtained from the guardians of all participants before specimens and data collection. Specimens were collected in accordance with Zhejiang Provincial CDC guidance and contained nasopharyngeal swab (n = 26), sputum (n = 3590) and bronchoalveolar lavage (n = 41). 2138 samples were from male and the remaining were from female. Clinical specimens were initially kept in sterile EP tube with 5 mL viral transport medium (Becton, Dickinson and Company, NJ) and then transferred to Zhejiang Provincial CDC within the same day for routine monitoring. All clinical samples were stored at − 80 °C prior to blinded analysis. Clinical data including symptoms, history of illness, severity of infection, demographic data, drug usage and laboratory examination were recorded for each patient and reviewed retrospectively when patients confirmed with HCoV infections.

### Nucleic acid extraction

Viral nucleic acid was extracted from 200 μL of clinical specimens using QIAGEN Cador Pathogen 96 QIAcube HT Kit (Qiagen, Hilden, German) and following manufacturer’s instructions. The extracts were eluted into 50 μL of DNase- and RNase-free water and stored at − 80 °C until used as a template for PCR assay.

### Two-tube multiplex reverse transcription PCR (RT-PCR) assay

A two-tube multiplex RT-PCR assay (a two-tube assay) was performed for the identification of sixteen respiratory virus using virus-specific primers as described previously [15]. Briefly, one tube was added with nine pairs of chimeric primers for the detection of nine viruses (HRV, FluA, FluB, 229E, OC43, HKU1, PIV1, PIV3 and ADV), and the other tube was added with eight pairs of chimeric primers to detect another eight viruses (RSVA, RSVB, NL63, PIV2, PIV4, H3N2, H1N1 and HBoV). The primer sequences, target genes, amplicon sizes and primer working concentrations were summarized in Additional file 1: Table 1. Qiagen one-step RT-PCR Kit (Qiagen, Hilden, German) was used for amplification. A final volume of 25 μL PCR mixture containing 2 μL of template, 1 μL of primer-mix (mixture was prepared according to the primer working concentration in Table 1), 1 μL of enzyme-mix, 1 μL of deoxinucleotidetriphospate (dNTP), 5 μL of 5X buffer, 1 μL of Taq polymerase was subjected to multiplex RT-PCR screening. The amplification cycle was initial denaturation at 50 °C for 30 min, 95 °C for 15 min, followed by 10 cycles of 95 °C for 30 s, 55 °C for 30 s, 72 °C for 30 s; 10 cycles of 95 °C for 30 s, 65 °C for 30 s, 72 °C for 30 s; 25 cycles of 95 °C for 30 s, 48 °C for 30 s, 72 °C for 30 s and one final extension at 72 °C for 3 min. The amplified fragment (15 μL) was separated on the QIAxcel automatic electrophoresis using QIAxcel DNA High-Resolution kit and confirmed as specific virus types following reference marker in the system.

**Table 1:** Distribution of 16 respiratory virus in children according to gender and age

	Total, n (%)	Gender, n (%)		Age, n (%)			
		Male	Female	0-1Y	1Y-2Y	2Y-10Y	10Y-18Y
*Sample*							
Total sample	3660	2138 (58.42)	1498 (40.93)	2694 (73.61)	311 (8.50)	552 (15.08)	79 (2.16)
Positive sample	1773 (48.44)	1091 (51.03)	673 (44.93)	1429 (53.04)	129 (41.48)	184 (33.33)	20 (25.32)
Human coronavirus (HCoV)	144 (3.93)	86 (4.02)	58 (3.87)	114 (4.23)	12 (3.86)	14 (2.54)	4 (5.06)
HKU1	38 (1.04)	24 (1.12)	14 (0.93)	31 (1.15)	1 (0.32)	5 (0.91)	1 (1.27)
NL63	62 (1.69)	35 (1.64)	27 (1.80)	47 (1.74)	7 (2.25)	6 (1.09)	2 (2.53)
OC43	38 (1.04)	25 (1.17)	13 (0.87)	33 (1.22)	4 (1.29)	1 (0.18)	0
229E	8 (0.22)	3 (0.14)	5 (0.33)	5 (0.19)	0	2 (0.36)	1 (1.27)
SARS-CoV-2	0	0	0	0	0	0	0
Influenza (Flu)	242 (6.61)	155 (7.25)	84 (5.61)	182 (6.76)	15 (4.82)	38 (6.88)	4 (5.06)
FluA	132 (3.61)	87 (4.07)	44 (2.94)	104 (3.86)	9 (2.89)	16 (2.90)	2 (2.53)
FluB	116 (3.17)	74 (3.46)	39 (2.60)	82 (3.04)	7 (2.25)	22 (3.99)	2 (2.53)
Respiratory syncytial virus (RSVs)	531 (14.51)	330 (15.43)	197 (13.15)	452 (16.78)	27 (8.68)	40 (7.24)	6 (7.59)
RSVA	366 (10.00)	223 (10.43)	142 (9.48)	307 (11.40)	25 (8.04)	23 (4.17)	6 (7.59)
RSVB	168 (4.59)	115 (5.38)	52 (3.47)	155 (5.75)	4 (1.28)	8 (1.45)	0
Parainfluenza (PIVs)	401 (10.96)	243 (11.37)	158 (10.55)	338 (12.55)	34 (10.93)	29 (5.25)	0
PIV1	37 (1.01)	28 (1.31)	9 (0.60)	30 (1.11)	3 (0.96)	4 (0.72)	0
PIV2	18 (0.49)	11 (0.51)	7 (0.47)	12 (0.45)	2 (0.64)	4 (0.72)	0
PIV3	338 (9.23)	204 (9.54)	134 (8.95)	286 (10.62)	28 (9.00)	24 (4.35)	0
PIV4	12 (0.33)	6 (0.28)	6 (0.40)	9 (0.33)	0	3 (0.54)	0
Human rhinovirus (HRV)	503 (13.74)	311 (14.55)	190 (12.68)	383 (14.22)	44 (14.15)	65 (11.78)	9 (11.39)
Human metapneumovirus (HMPV)	111 (3.03)	59 (2.76)	52 (3.47)	101 (3.75)	4 (1.29)	5 (0.91)	0
Human bocavirus (HBoV)	63 (1.72)	34 (1.59)	28 (1.87)	43 (1.60)	12 (3.86)	7 (1.27)	0
Human adenovirus (ADV)	140 (3.83)	89(4.16)	51 (3.40)	92 (3.41)	17 (5.47)	29 (5.25)	2 (2.53)

The specificity of the two-tube assay was based on the automatic electrophoresis. All positive controls were observed and separated clearly from the other viral targets and no primer dimer was observed in either tube. The expected size of each virus type/subtype-specific amplicon was listed in Additional file 1: Table 1. The detection sensitivity in tube 1 and tube 2 was 2000 and 200 copies per reaction, respectively. The detection limit for HRV, PIV2, PIV3, RSVA, HBoV and ADV was 20 copies per reaction and the remaining 10 viruses had a detection limit of 200 copies per reaction.

### Detection of SARS-CoV-2 by RT-qPCR

SARS-CoV-2 nucleic acid detection kit (Daan, China) was applied to detect the ORF1ab gene and N gene of SARS-CoV-2 according to protocols as described previously [16]. Primers and probes used for SARS-CoV-2 were listed in Additional file 1: Table 1. The reaction was performed in a 25 μL reaction mixture containing 17 μL of reaction mixture A, 3 μL of reaction mixture B and 5 μL of RNA. The RT-qPCR reactions were performed with initial one cycle at 50 °C for 15 min and 95 °C for 15 min, following by 45 cycles of 94 °C for 15 s and 55 °C for 45 s. All RT-qPCRs were performed on the 7500 Fast Real-Time PCR system (Applied Biosystems) and all samples were analyzed in duplicate.

### Sequencing of spike (S) gene

The S genes of HKU1, OC43, NL63 and 229E positive samples were amplificated using PrimeScript™ One Step RT-PCR Kit Ver.2 (TaKaRa 055RA). The volume of PCR mixture was 25 μL, containing buffer 12.5 μL, enzyme-mix 1 μL, forward primer 0.5 μL, reverse primer 0.5 μL, RNA 5 μL, and Rnase free water 5.5 μL. Their forward and reverse primers were listed in Additional file 1: Table 2. The amplification cycle was reverse transcription at 50 °C for 30 min and initial denaturation at 94 °C for 2 min, followed by 40 cycles of 94 °C for 45 s, 50–56 °C (depending on species) for 30 s, 72 °C for 45 s, and one final extension at 72 °C for 10 min at the end of 40 cycles. Amplified PCR products were sequenced by Sanger sequencing method (TsingKe Biological Technology).

**Table 2:** Viral detection rate according to sample type

Virus	Total, n (%)	Nasopharyngeal swab, n (%)	Bronchoalveolar lavage, n (%)	Sputum, n (%)
*Sample*				
Total sample	3660	26 (0.71)	41 (1.12)	3590 (98.09)
Positive sample	1773 (48.44)	13 (50.00)	22 (53.66)	1738 (48.41)
Human coronavirus (HCoV)	144 (3.93)	1 (3.85)	2 (4.88)	141 (3.93)
HKU1	38 (1.04)	0	0	38 (1.06)
NL63	62 (1.69)	1 (3.85)	2 (4.88)	59 (1.64)
OC43	38 (1.04)	0	0	38 (1.06)
229E	8 (0.22)	0	0	8 (0.22)
SARS-CoV-2	0	0	0	0

### Phylogenetic analysis

DNA sequences were concatenated manually and aligned by Clustal W and downloaded to MEGA 7.0 to generate a phylogenetic tree constructed by neighbor-joining algorithm and jukes-cantor model with 1000 bootstrap. Genome sequences of other coronaviruses were retrieved from GeneBank and included in phylogenetic analysis for comparison.

### Statistical analysis

The statistical software SPSS (Statistics 20, IBM, Armonk, NY) was used for data processing and statistical analysis. Fisher’s Chi-square test was performed to test for statistically significant associations between prevalence of human coronaviruses to season, age, patient gender, other respiratory viruses, and clinical sample type. *P* values of < 0.05 were considered as significant. Analyses of variance (ANOVAs) were also performed to test the null hypothesis that there was no difference between clinical manifestations and HCoVs infection. If a null hypothesis was rejected at the 0.05 level, a Tukey’s multiple-comparison test was used to identify differences in clinical characteristics.

## Results

### Prevalence of four human coronaviruses in Zhejiang Province, China

**Fig. 1:**
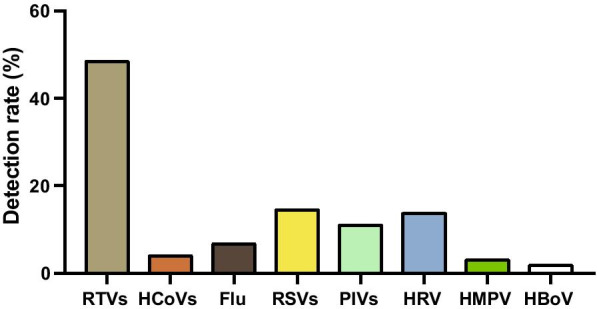
The detection rates of respiratory viruses

To survey human coronaviruses (HCoVs) in Zhejiang province, nasopharyngeal swabs (n = 26), sputum (n = 3593) and bronchoalveolar-lavage fluid (n = 41) specimens were collected periodically between February 2017 and December 2019 from children with respiratory symptoms. Overall, all clinical specimens were negative for SARS-CoV-2 RNA by RT-PCR analysis. 38 (1.04%) were positive for HCoV-HKU1, 62 (1.69%) for HCoV-NL63, 8 (0.22%) for HCoV-229E and 38 (1.04%) for HCoV-OC43 (Table 1). Comparison of the percentage of positive samples for multiple respiratory viruses was used to infer prevalence (Fig. 1). Respiratory syncytial viruses (RSVA + RSVB, 14.51%), human rhinovirus (HRV, 13.74%), parainfluenza (PIVs 1–4, 10.96%) and influenza (FluA + FluB, 6.61%) were the four dominant viruses in Zhejiang area, which were detected more frequently than HCoVs (*P* < 0.001).

### Circulation trend

**Fig. 2:**
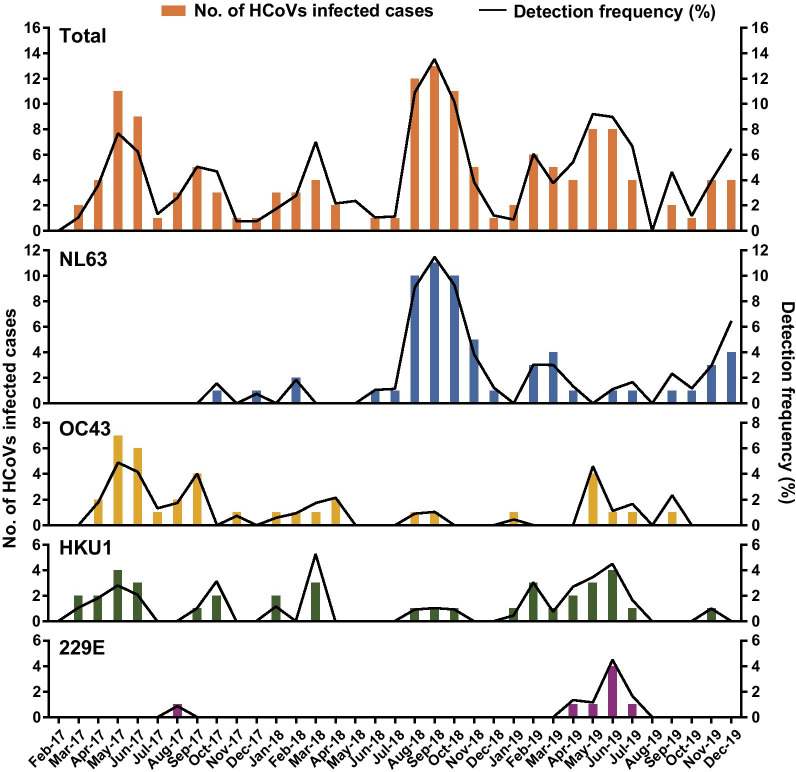
Monthly distribution of total HCoVs (orange), HCoV-NL63 (blue), HCoV-OC43 (yellow), HCoV-HKU1 (green) and HCoV-229E (purple) infections in hospitalized children with respiratory tract infections from February 2017 to December 2019

**Fig. 3:**
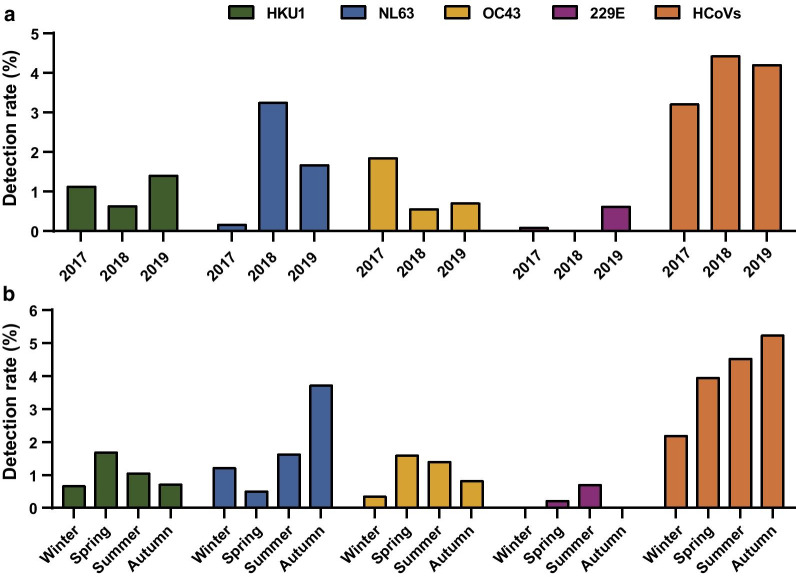
Circulation trends of HCoV infections. **a** Yearly distribution of the four HCoV infections. **b** Seasonal distribution of the four HCoV infections

Overall, HCoVs dominant circulation season was in the transition of summer to fall, especially August to October (Fig. 2). The percent of HCoV positive tests varied seasonally throughout each year and the percent positive also varied annually by HCoV species (Fig. 3a). The detection rates of HCoVs were 2.18%, 3.94%, 4.51%, 5.22% of winter, spring, summer and autumn, respectively (Fig. 3b). HCoV-HKU1 demonstrated a distinct peak each of the three years, with a relatively large peak in Spring 2018. For HCoV- NL63, in contrast to the pattern described for HCoV-HKU1, the peak detection frequency occurred in the third season. 51.61% of positive HCoV- NL63 samples were detected between September to November. HCoV-OC43 also demonstrated a consistent detection frequency between seasons, with the testing peak occurring each year in spring. The exception was HCoV-229E, as 87.50% of detectable HCoV-229E were found in the spring and summer of 2019. To be noted, HCoV-229E detected only in spring and summer through the study period, and no HCoV-229E was detected in 2018.

### Clinical profiles associated with HCoV infections

Almost all HCoV-positive patients (143/144, 99.31%) had lower respiratory tract infections. The incidence of lower respiratory tract infections in patients with HCoVs (3.94%) infection was significantly lower than that of patients infected with RSV (14.51%), PIV (10.96%), or HRV (13.85%). In addition, only one patient infected with HCoV-NL63 was diagnosed with an upper respiratory tract infection from nasopharyngeal swab in this study. The positive rate of HCoV infections was 3.85% (1/26) for nasopharyngeal swab, 4.88% (2/41) for bronchoalveolar lavage and 3.93% (141/3590) for sputum (*P* > 0.05) (Table 2). The four investigated HCoV subtypes were detected in sputum samples, while only HCoV-NL63 infections were positive in nasopharyngeal swab and bronchoalveolar lavage specimens.

**Fig. 4:**
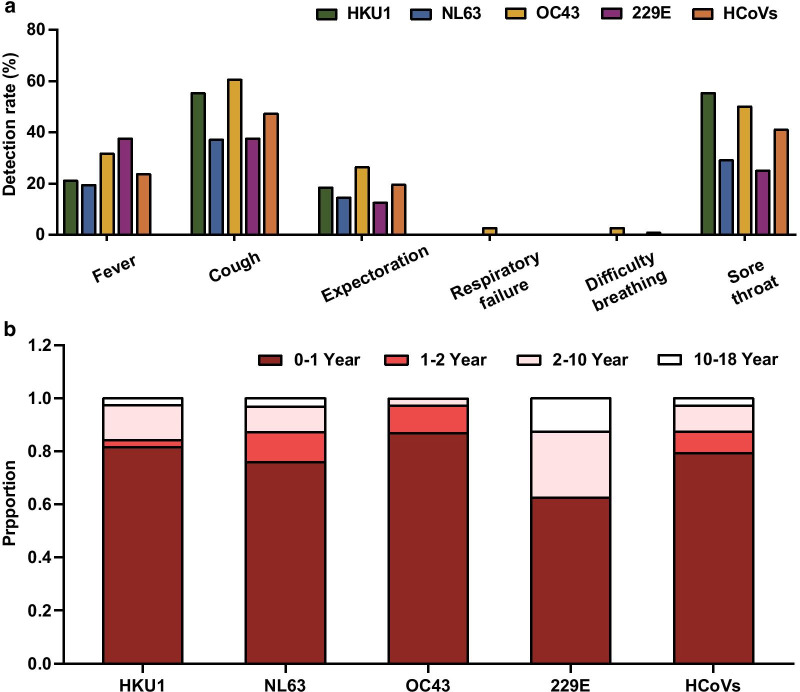
Clinical profiles of HCoV infections based on clinical manifestation (**a**) and age (**b**)

Fever, cough, expectoration and sore throat were the common clinical presentations with HCoV infections, among which cough (47.22%), sore throat (40.97%) and fever (23.61%) were the top three manifestations (Fig. 4a). Difficulty in breathing and respiratory failure were seen in patients who infected with HCoV-OC43. Slightly more patients with HCoV infections were males but no significant difference was in the sex distribution between the four species (*P* > 0.2) (Table 1). Age distribution of patients differed between HCoV species and infants (< 1 year old) with weak respiratory immunity were most susceptible to four types of HCoVs infections (Fig. 4b). To be noted, HCoV-229E had a higher detection proportion in children between 2 and 18 years old compared to HCoV-OC43, HCoV-NL63, and HCoV-HKU1.

### Coinfection of respiratory viruses

High rates of coinfection with other respiratory viruses were observed for all coronaviruses, with the coinfection rates of HCoV-HKU1, HCoV-NL63, HCoV-OC43 and HCoV-229E as 44.74% (17/38), 43.55% (27/62), 52.63% (20/38) and 37.50% (3/8), respectively (Table 3). Among the 146 positive detections, 54.11% reported a single HCoV species detection only, 1.37% reported two HCoV species, and 44.52% detected another respiratory virus. The most common HCoV co-infections were HCoV-HKU1 with HCoV-229E (2 specimens, 1.37%). Of all samples testing positive for coronavirus, 40% samples were coinfected with HRV. The most common co-detected non-HCoVs were human adenovirus (40.71%), human rhinovirus/bocavirus (37.57%), and human metapneumovirus (32.43%).

**Table 3:** Coinfection of 16 respiratory virus

Virus	Number of cases												Rate (%)
	HKU1	NL63	OC43	229E	SARS-CoV-2	Flu	RSVs	PIVs	HRV	HMPV	HBoV	ADV	
HKU1	38	0	0	2	0	0	4	5	4	0	2	0	1.04
NL63		62	0	0	0	5	4	2	16	1	2	1	1.69
OC43			38	0	0	3	5	7	6	2	1	2	1.04
229E				8	0	0	0	1	0	0	0	0	0.22
SARS-CoV-2					0	0	0	0	0	0	0	0	0
Flu						242	18	14	11	2	2	10	6.61
RSVs							531	26	70	5	4	12	14.51
PIVs								401	51	10	2	13	10.96
HRV									503	10	9	13	13.74
HMPV										111	1	5	3.03
HBoV											63	1	1.72
ADV												140	3.83
One virus	21	35	18	5	0	181	399	275	314	75	39	83	39.48
Two viruses	17	23	14	3	0	52	113	109	166	29	19	43	8.03
Three viruses	0	4	6	0	0	9	18	16	22	7	5	13	0.90
Four viruses	0	0	0	0	0	0	1	1	1	0	0	1	0.03

### Phylogenetic analysis of HCoV strains

**Fig. 5:**
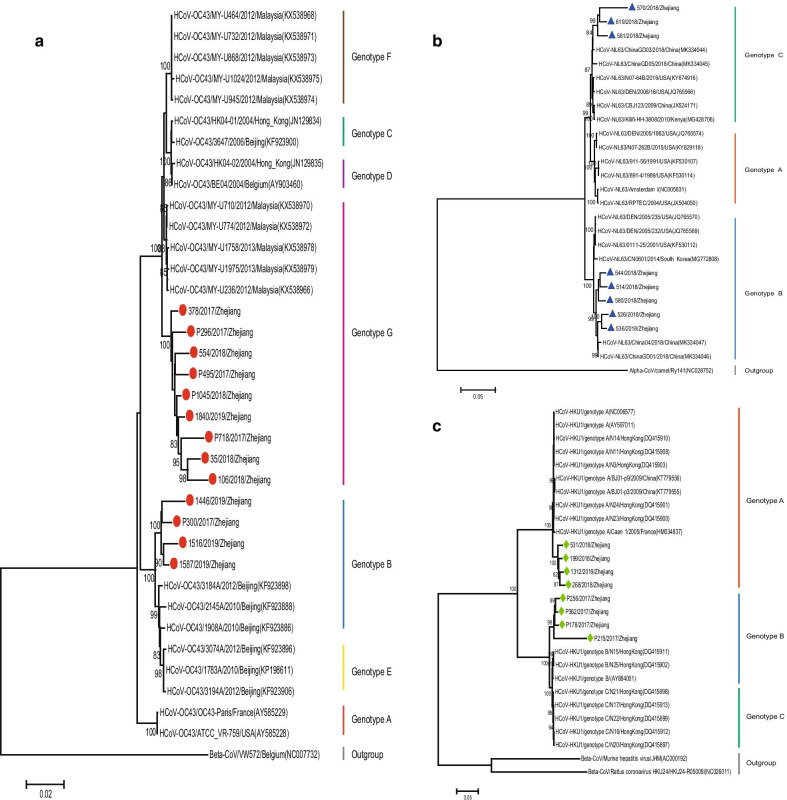
Phylogenetic analysis based on nucleotide sequences of PCR products corresponding to the spike genes of HCoV-OC43, HCoV-NL63 and HCoV-HKU1. **a** Phylogenetic trees of HCoV-OC43 S gene (4.7 kb) constructed with neighbor-joining algorithm. Red circles indicated Zhejiang HCoV-OC43 strains. **b** Phylogenetic trees of HCoV-NL63 S gene (4.5 kb) constructed with neighbor-joining algorithm. Blue triangles indicated Zhejiang HCoV-NL63 strains. **c** Phylogenetic trees of HCoV-HKU1 S gene (4.6 kb) constructed with neighbor-joining algorithm. Green rhombus indicated Zhejiang HCoV- HKU1 strains

Phylogenetic trees were further constructed based on the sequencing of HCoV spike genes. A representative subset of HCoV-OC43 (n = 13), HCoV-NL63 (n = 8) and HCoV-NL63 (n = 8) were included in the phylogenetic analysis and compared to the HCoVs from clinical sources worldwide. A high level of genetic diversity was observed among those HCoVs. The OC43 coronaviruses were clustered into genotype G (9, 69.23%) and genotype B (4, 30.77%) and related to the sequences detected in Malaysia and Beijing, respectively (Fig. 5a). No significant differences were observed between HCoV-OC43 strains identified in different years. Similarly, HCoV-NL63 strains in this study were clustered into genotype B (5, 62.5%) and genotype C (3, 37.5%) and shown close identity to the viruses isolated from China and USA (Fig. 5b). Moreover, the phylogenetic tree of the HCoV-HKU1 isolates shown the existence of two major clusters that contained both Hong Kong and France strains of HCoV-HKU1 (Fig. 5c). All HCoV-HKU1 subtypes isolated from 2017 were grouped into genotype B.

## Discussion

Emerging and reemerging pathogens represent a serious burden to global health. The recent emergence of SARS-CoV-2 clearly demonstrated the epidemic potential of coronaviruses. Here we demonstrated that HCoV-OC43, HCoV-NL63, HCoV-HKU1 and HCoV-229E were specifically associated with lower respiratory tract disease in pediatric population, accounting for 3.93% of all admissions for acute respiratory infections. Only one patient was associated with upper respiratory traction infection from nasopharyngeal swab samples. The newly discovered SARS-CoV-2 RNA was absent in all tested samples, suggesting no introduction of this highly pathogenic coronavirus into the Zhejiang population before 2020. RSV (14.51%) was the predominant respiratory virus in our pediatric population. Our results aligned with studies conducted in other countries whereby RSV was the predominant viral agent among young children [17–20]. All the HCoVs detected have been subtyped. HCoV-NL63 (1.69%) was the most prevalent coronaviruses in our study consistent with reports in Hong Kong [21], Japan [22], Brazil [23] and Kenya [24], but some studies demonstrated the occurrence of HCoV-OC43 was similar or even higher than that of HCoV-NL63 in United States [9], United Kingdom [25] and Guangdong province [26].

In general, common HCoVs are reported to have annual peaks of circulation in winter months and few detections in summer months in temperate regions [9, 22, 25, 27]. Our data shown a completely different trend as we found HCoVs had a summer-autumn peak of activity, suggesting seasonality of HCoVs in subtropical regions was not restricted to the winter. Individual HCoV subtypes shown variable circulation throughout each year. During the sampling period of this study, HCoV-OC43 was the major circulating strain in 2017, whereas HCoV-NL63 predominated in the following two years. This was expected, given previous observations of annual variation in the circulation of HCoVs in Japan [22], United States [9] and Thailand [28]. HCoV-229E was only detected in spring and summer with a comparatively low detection frequency. This reaffirmed with the report in United Kingdom indicating that HCoV-229E was lacking a discernible seasonality [25]. However, continuous surveillance of HCoV infections over a long period is required to delineate their circulation trend more precisely.

This study investigated children between age below 18 years old. The burden of HCoV infections in children was high, especially among infants ((< 1 year old) because of their immature immune system. The age distribution of patients varied between HCoV species. Similar to the epidemiological feature in United States [9], HCoV-OC43 detections were most prevalent in infants, while HCoV-229E infections were most common among elder children. In concordance with a previous study [17], we have also found that boys were more vulnerable (4.02%) to HCoV infections than girls (3.87%). Specimen types are important for the diagnosis of respiratory viral infections. Although the overall detection rate of HCoVs was similar between sample types, sputum was superior to bronchoalveolar lavage and nasopharyngeal swab as all HCoV subtypes were detected from sputum. This finding was consistent with previous reports that sputum was more sensitive than nasopharyngeal swabs for respiratory viral detection [29, 30]. Although more than half one-third of the HCoV positive samples were co-detected with other viruses, our data support that HCoVs lead to a substantial burden of respiratory tract infections in children.

Phylogenetic analysis based on the spike gene confirmed previously published results and illustrated the genetic diversity of HCoV strains. Recombination occurred in HCoV frequently. The primary circulating genotypes of HCoV-OC43 in our study were B and G, while none of the genotype A, C, D, E and F were detected. This finding was consistent with Guangzhou strains, where their dominant circulating genotypes were B and G [31]. The phylogenic tree based on spike gene of HCoV-NL63 revealed the genotypes circulating in Zhejiang area were B and C, which was different from Kenya as their circulating genotypes were A and B [32]. However, this database was too small to reflect the actual variation level of HCoVs. Further research is needed to apply whole genome sequencing as the primary sequencing method for the genetic characterization of HCoVs.

To best of our knowledge, this comprehensive survey is the first to describe the clinical patterns and genetic characterization of the four major HCoVs and SARS-CoV-2 in Zhejiang province during a multi-year period. However, some limitations still existed to this survey. We only evaluated respiratory symptoms among hospitalized children, while the data from asymptomatic children or patients with mild respiratory symptoms were not included. Additionally, we assessed HCoV percent positivity based on the submission of specimen collection. Aggregate data might contain multiple specimens from the same patient, potentially affecting the demographic characteristics of our total study populations. Therefore, our data probably had some bias to represent the overall health burden of HCoVs infections of the general pediatric population in Zhejiang province, China.

## Conclusion

Taken together, this study revealed that human coronaviruses were a significant cause of acute respiratory infections among hospitalized children, especially infants. Individual HCoVs may show variable circulation from year to year. As the global COVID-19 outbreak continues to grow, more investigations and rigorous surveillance are warranted to ascertain the circulation patterns and molecular epidemiology of human coronaviruses in China.

## Supplementary information

**Additional file 1.** **Supplementary table 1.** Sequences of oligonucleotides used for molecular analysis.

## Data Availability

The datasets used and analyzed during the current study are available from the corresponding author on reasonable request.

## Abbreviations

HCoVs: Human coronaviruses
RNA: Ribonucleic acid
SARS-CoV: Severe acute respiratory syndrome coronavirus
SARS-CoV-2: Severe acute respiratory syndrome coronavirus 2
MERS-CoV: Middle east respiratory syndrome coronavirus
HCoV-NL63: Human coronavirus NL63
HCoV-OC43: Human coronavirus OC43
HKU1: Human coronavirus HKU1
HCoV-229E: Human coronavirus 229E
PCR: Polymerase chain reaction
RSVs: Respiratory syncytial viruses
RSVA: Respiratory syncytial virus A
RSVB: Respiratory syncytial virus B
PIV1: Parainfluenza virus 1
PIV2: Parainfluenza virus 2
PIV3: Parainfluenza virus 3
PIV4: Parainfluenza virus 4
HRV: Human rhinovirus
FluA: Influenza virus A
FluB: Influenza virus B
H3N2: FluAInfluenza virus H3N2
H1N1: Influenza virus H1N1
HBoV: Human bocavirus
ADV: Adenovirus
