# Supplementary material for: Etiology and clinical characteristics of SARS-CoV-2 and other human coronaviruses among children in Zhejiang Province, China 2017–2019
Source: Virol J. 2021 Apr 30;18:89. doi: 10.1186/s12985-021-01562-8 (PMC8085659; doi:10.1186/s12985-021-01562-8)
Supplement: Supplementary file 1 — Additional file 1. Supplementary table 1. Sequences of oligonucleotides used for molecular analysis. [file 12985_2021_1562_MOESM1_ESM.pdf]

Supplementary table 1

Supplementary table 1 Sequences of oligonucleotides used for molecular analysis.

Two-Tube Assay	Virus	primer	Target gene	Primer set 5'-3'	Size (bp)	Concentrations*
Tube 1						
	HRV	HRV-F	5'-UTR	<u>GTACGACTCACTATAGGGA</u>CCCCTGAATGYGGCTAACCT	145	75 nM/L
		HRV-R		<u>GTACGACTCACTATAGGGA</u>CGGACACCCAAAGTAGTYGGT		
	FluB	FluB-F	M	<u>GTACGACTCACTATAGGGA</u>AAAAGRAGATTCATCACAGAGC	167	50 nM/L
		FluB-R		<u>GTACGACTCACTATAGGGA</u>TTCTGCTATTTCAAATGCTTCA		
	229E	229E-F	N	<u>GTACGACTCACTATAGGGA</u>CTCGGAATCCTTCAAGTGACAGA	184	50 nM/L
		229E-R		<u>GTACGACTCACTATAGGGA</u>ACGAGAAGGCTTAGGAGTAC		
	OC43	OC43-F	N	<u>GTACGACTCACTATAGGGA</u>ATTGCACCAGGAGTCCCA	200	50 nM/L
		OC43-R		<u>GTACGACTCACTATAGGGA</u>TATCGGTGCCGTACTGGTCT		
	HKU1	HKU1-F	N	<u>GTACGACTCACTATAGGGA</u>TATAGTRAAACCTGATATGGCT	221	50 nM/L
		HKU1-R		<u>GTACGACTCACTATAGGGA</u>TACCAAAACACTGTTGAACAT		
	PIV3	PIV3-F	HA	<u>GTACGACTCACTATAGGGA</u>TTGTCAATTATGATGGYTCAATCT	236	50 nM/L
		PIV3-R		<u>GTACGACTCACTATAGGGA</u>TTTTTGACACCCAGTTGTGTTGCAG		
	FluA	FluA-F	M	<u>GTACGACTCACTATAGGGA</u>CATTGGGATCTTGCACTTGATATT	265	100 nM/L
		FluA-R		<u>GTACGACTCACTATAGGGA</u>TTTTTTTTTGTAGAAACAAGGTAGTTTTTTACTC		
	PIV1	PIV1-F	HA	<u>GTACGACTCACTATAGGGA</u>TCTCATTATTACCYGGACCAA	285	87.5 nM/L
		PIV1-R		<u>GTACGACTCACTATAGGGA</u>TCCTGTTGTCGTTGATGTCATA		
	ADV	ADV-F	Hexon	<u>GTACGACTCACTATAGGGA</u>GCCSCARTGGKCWTACATGCACATC	339	100 nM/L
		ADV-R		<u>GTACGACTCACTATAGGGA</u>CAGCACSCCICGRATGTCAAA		
Tube 2						
	RSVA	RSVA-F	F	<u>GTACGACTCACTATAGGGA</u>CATCCCCTCTATGCACAACC	159	50 nM/L

	RSVA-R		GTACGACTCACTATAGGGACATGTTTCAGCTTGTGGGAA		
NL63	NL63-F	Polymerase	GTACGACTCACTATAGGGATCCCAAATGTGATAGAGCTTTGC	177	50 nM/L
	NL63-R		GTACGACTCACTATAGGGACTGTTAAAACTTGTGCCAACTC		
PIV2	PIV2-F	HA	GTACGACTCACTATAGGGATCTACACTGCATCAGCCAGC	195	50 nM/L
	PIV2-R		GTACGACTCACTATAGGGACCCCTAAAAGAGATGAGCCC		
HMPV	HMPV-L-F	L	GTACGACTCACTATAGGGACATGCCCACTATAAAAGGTCAG	209	100 nM/L
	HMPV-L-R		GTACGACTCACTATAGGGACACCCCAGTCTTTCTTGAAA		
	HMPV-N-F	N	GTACGACTCACTATAGGGAGAGCTAAYAGAGTGCTAAGTGATG	209	50 nM/L
	HMPV-N-R		GTACGACTCACTATAGGGAACTTTCTGCTTTGCTTCCTGT		
RSVB	RSVB-F	F	GTACGACTCACTATAGGGAAAACGAAGATTTCTGGGCTTC	280	75 nM/L
	RSVB-R		GTACGACTCACTATAGGGATGCGACAGCTCTGTTGATTT		
HBoV	HBoV-F	NP1	GTACGACTCACTATAGGGAAAGAAAAGGGAGTCCAGAA	298	100 nM/L
	HBoV-R		GTACGACTCACTATAGGGATTTTTTTTCTCTGTGTTGACTGAATACAG		
PIV4	PIV4-F	Phosphoprot	GTACGACTCACTATAGGGACTGAACGGTTGCATTCAGGT	480	50 nM/L
	PIV4-R	ein	GTACGACTCACTATAGGGATTGCATCAAGAATGAGTCCT		
Internal control primer					
Rnasep	Rnasep F	RNase P	GTACGACTCACTATAGGGAGAGGCCTGGCTTTTGAACTT	126	25 nM/L
	Rnasep R		GTACGACTCACTATAGGGAATCAAATTGAGGGCACTGGA		
Universal primer					
	Tag		GTACGACTCACTATAGGGA		10 μM/L

* Varied primer concentrations in 25 μL PCR mixture.

Supplementary table 2

Supplementary table 2 Primer of S genes for HKU1, HCoV-NL63 and HCoV-OC43.

Primer	Sequence 5'-3'	Location
HKU1 F1	GCTGTTATAGGTGATTTTAATTG	23150-23170
HKU1 R1	CGTTCCCAATTAAGAGGTGA	24130-24150
HKU1 F2	CCTAATACTGGTGTTTATGA	24010-24030
HKU1 R2	CAATCAATAGTAACTTTAGGAG	25550-25570
HKU1 F3	CAAGGTATTTTTAAAGAAGTTTCTG	25010-25030
HKU1 R3	CTATTAAGTGCTTGAGCATTAG	26240-26260
HKU1 F4	GTTTGGGTGTTACTATGGATGTTC	26100-26110
HKU1 R4	CATACTCATCACAACAATTATGAC	27130-27150
NL63 F1	GAGTGGTAGGTTGTTGTTACG	20520-20540
NL63 R1	CCCACAAAAGTGCTAACATGAG	21670-21690
NL63 F2	GCCACTGGTTCTGATGTTAATTG	21420-21440
NL63 R2	CTGGTTGGTTACATGGTGTCAC	22610-22630
NL63 F3	CGTTCTTCAAACCAGTCACTTGC	22520-22540
NL63 R3	GCCTTATTAAATGATGCAGCC	23520-23540
NL63 F4	CTTTGGCACTGCAAGCACGAC	23440-23460
NL63 R4	GAGTAAGTTGAAATAGGCCACC	24670-24700
OC43 F1	CTAGGCTGCATGATGCTTAGACC	23710-23730
OC43 R1	GACATCAGGCTGCTCATATT	24860-24880
OC43 F2	AACACAATCTAT AGCACCACC	24680-24700
OC43 R2	CATATAAAAGGTTCTGCCAAC	25720-25740
OC43 F3	GGCATTTTTGTTGAGGTTAATGCG	25680-25700
OC43 R3	CCAAATCTATTAGAGAGTTGTTGC	26920-26950
OC43 F4	CCAGTAGATCTGCTATAGAGG	26490-26510
OC43 R4	GACGAACTTAGTCGTCATGTGAAG	27830-27850
